# Metropolitan Provident Medical Association

**Published:** 1899-06-17

**Authors:** 


### METROPOLITAN PROVIDENT MEDICAL ASSOCIATION.

The annual report of the Metropolitan Provident Medical Association shows a very satisfactory year's work as having been done. The number of membership cards, as compared with 10,525 in the previous year, was 11,441, which brought the total number of members to over 34,000. The provident members' payments, amounting to £5,304, showed an increase of £466. The receipts for the year totalled £6,368, while the expenditure was £6,357. Subscriptions and donations showed a falling off of £53 since 1897. The number of examinations of patients made during the year was 1,080, and 797 letters according the privilege of treatment at the dispensaries, were issued. The affiliation of the Blackfriars Provident Dispensary to the association is recorded. Ths hospitals, the report says, have shown a friendly disposition towards the association, and some have co-operated with them to the extent of posting notices having reference to the provident dispensaries, and of allowing the recommendation of these to patients by the inquiry officers. The Woolwich, Whitechapel, and the new Leman Street branches are all making satisfactory progress.

The annual drawing-room meeting of the association was held at the residence of Mr. Denny and Lady Hope on Tuesday, the 6th inst., the chair being taken by Mr. Denny. Among those present were the Dean of St. Paul's, Sir William Broadbent, Lord Fortescue, Sir Joshua Fitch, the Rev. Harry Jones, and Mr. Samuel Lewes. The proceedings opened with the reading by the Secretary of letters received from the Duke of Westminster, Lord Stamford, Lord Lonsdale, Mr. Claude Montefiore, and others, expressing regret at their inability to attend, and after introductory remarks by the Chairman, Sir William Broadbent moved a resolution to the effect that the establishment of provident dispensaries, co-operating with the hospitals, and under rules approved by the medical profession, would be for the benefit of the working classes. In the course of his speech Sir William remarked that it was not the very poorest nor the class immediately above them, but the prosperous working-class for which there was difficulty in providing suitable medical attendance. This difficulty was done away with by the establishment of benefit clubs and provident dispensaries. From these they could receive help without losing their self-respect. In the treatment of the sick it was not so much physic as advice that was of importance. This, the speaker observed, could not be given without a knowledge of the surroundings, family, and habits of the patient. The provident dispensaries had this great advantage over the hospital out-patient departments, that confidential relations, which were of immense help to successful treatment, were established between doctor and patient. In the out-patient departments this was impossible. Referring to the co-operation of the hospitals, Sir William said that cases of a complicated nature requiring special operative skill were of occasional occurrence, and he was of opinion that the fact of membership with the association instead of forming, as it did, a cause for reluctant reception at the hospitals should be a claim to priority of treatment. He hoped for more direct future relations between the hospitals and provident dispensaries. The resolution was seconded by Lady Hope, who made a pleasantly humorous and sympathetic speech, which was well received. Mr. C. S. Loch, in supporting the resolution, said the existing system of medical relief had great need of reformation. There was a vital necessity for keeping up among the working-classes the spirit of self-reliance, which the work of the hospitals, unless great care were exercised, had a tendency to destroy. The motion having been carried, a resolution expressing the satisfaction of those present at the progress made by the association was then moved by the Dean of St. Paul's. The resolution was carried, and a vote of thanks to Mr. Denny and Lady Hope terminated the proceedings. Despite the reference to co-operation with the hospitals there was observable throughout the meeting a subdued note of antagonism with the hospitals, which in one or two cases found vent in ironical references to balls and bazaars as a means of obtaining funds for these charities.

